# GENERALIZED ERUPTIVE SYRINGOMAS

**DOI:** 10.4103/0019-5154.48992

**Published:** 2009

**Authors:** Mahnaz Jamalipour, Mitra Heidarpour, Parvin Rajabi

**Affiliations:** *From the Department of Dermatology, Najafabad Azad University, Isfahan, Iran*; 1 *From the Department of Pathology, Isfahan University of Medical Sciences, Isfahan, Iran*

**Keywords:** *Generalized eruptive syringoma*, *eccrine ducts*, *papules*

## Abstract

Generalized eruptive syringoma is a rare clinical presentation of a benign adnexal tumor that derives from the intraepidermal portion of the eccrine sweat ducts. It presents as successive crops of small flesh-colored papules on the anterior body surfaces. It generally occurs in the peripubertal period. Treatment of this benign condition is cosmetic only. A case of a 28-year-old female with an eight-year history of eruptive syringoma is presented.

## Introduction

Syringomas are benign adnexal tumors of eccrine origin, with four principal clinical variants.[1] In eruptive syringoma, a rare variant first described by Jacquet and Darier in 1987, the lesions occur in large numbers and in successive crops on the anterior chest, neck, upper abdomen, axillae, and the periumbilical region at puberty or during childhood.[2] More rarely, cases with wider involvement of the body have also been reported.[3] It occurs more frequently among women. The lesions consist of asymptomatic multiple small firm yellow-brown-colored papules, that typically present in a bilateral, symmetrical distribution, but there have been reports of unilateral, unilateral nevoid, bathing trunk and generalized distributions.[4,5] The lesions are benign and may spontaneously resolve, or, more commonly, remain stable. Treatment of this benign condition is cosmetic only.[3] Clinically, it may be mistaken for acne vulgaris, sebaceous hyperplasia, milia, lichen planus, eruptive xanthoma, urticaria pigmentosa, hidrocystoma, trichoepithelioma and xanthelasma on the face and granuloma annular on the trunk. Definitive diagnosis can be made on histological examination, because syringomas demonstrate distinctive histopathological features.[6]

## Case Report

A 28-year-old white female presented with an eight-year history of a dermatosis involving the upper extremities, axillae, and the anterior chest. The patient was admitted to a private dermatology clinic in Isfahan, with a generalized eruption with mild pruritus during perspiration, of eight-year duration. The lesions appeared on the forearm first, which was followed by successive eruptions on the face, chest, upper abdomen, thigh and neck respectively. The patient denied any medical problems or the use of over-the-counter preparations. The review of systems was noncontributory. The lesions were treated as common wart, but they did not resolve. There was no one in the family who was similarly affected. There was no spontaneous resolution in the period of observation.

**Figure 1:**
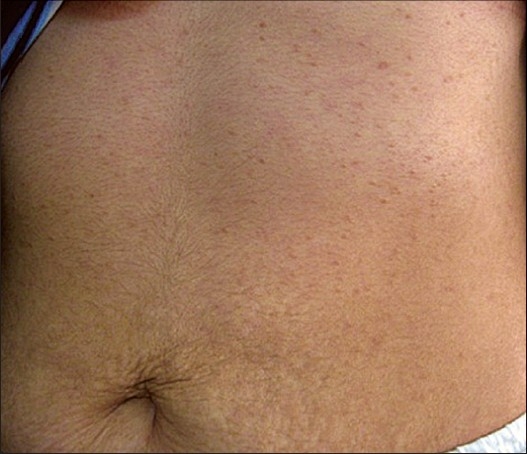
Multiple skin-to tan-colored flat topped papules on the upper abdomen and anterior chest

**Figure 2:**
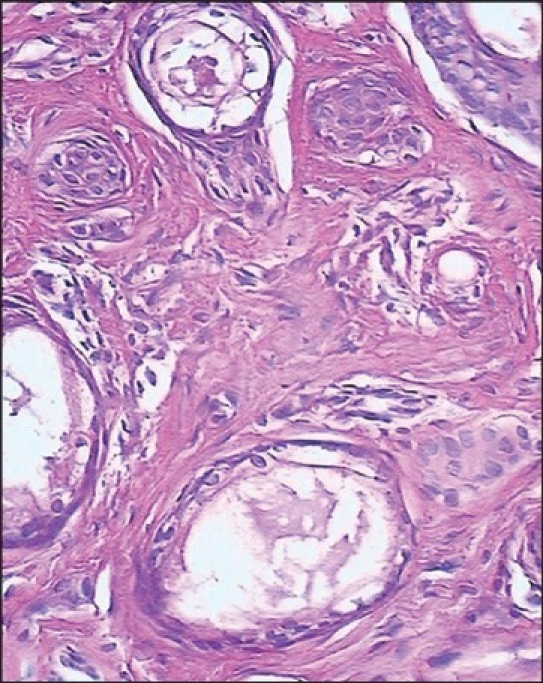
Section showing collections of cystic ducts and some epithelial cords with comma-like tails (H & E, ×240)

**Figure 3:**
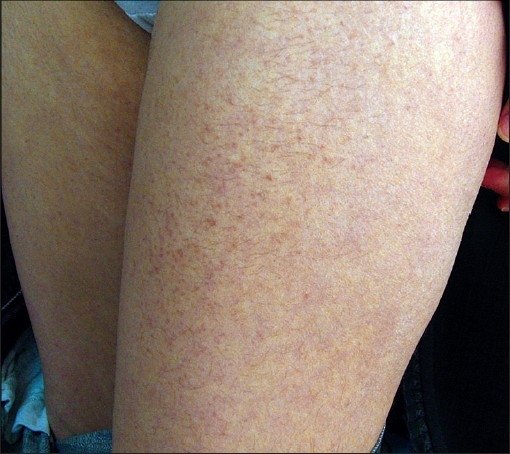
Multiple skin- to tan-colored flat topped papules on the thighs

**Figure 4:**
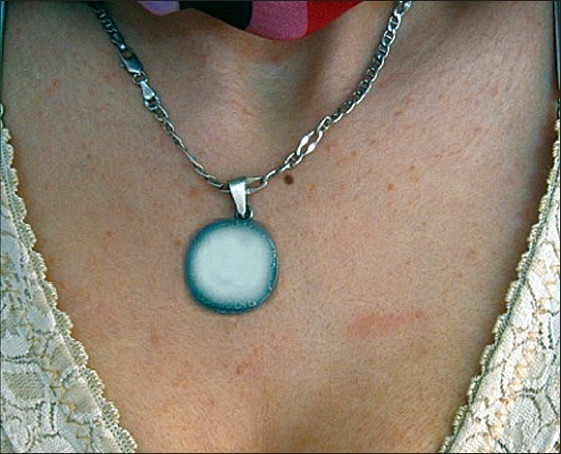
Multiple skin- to tan-colored flat topped papules on the anterior chest

**Figure 5:**
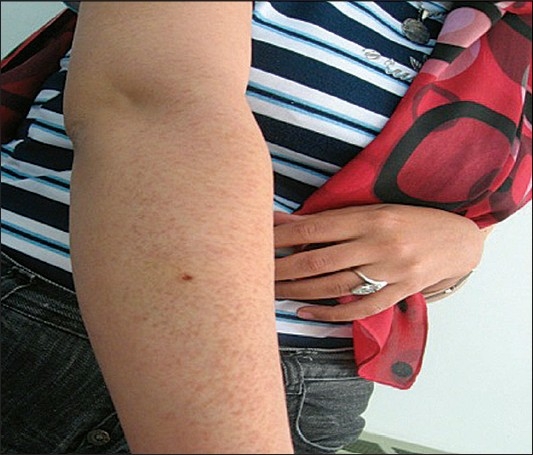
Multiple skin- to tan-colored flat topped papules on the forearm

**Figure 6:**
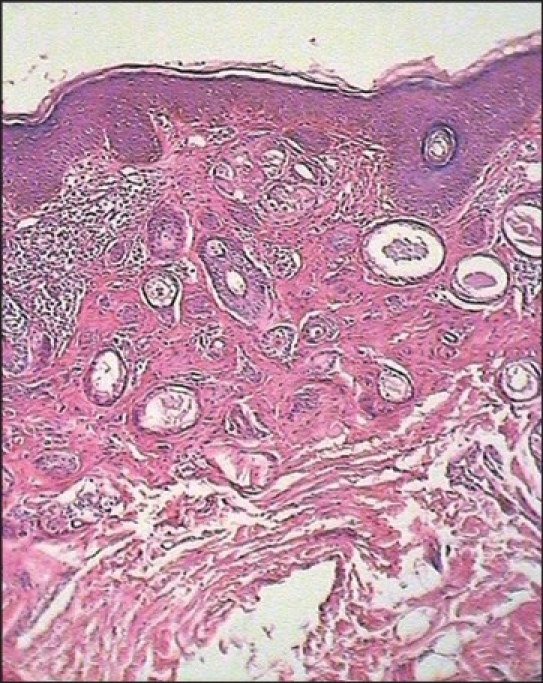
Section, showing collections of cystic ducts and some epithelial cords with comma-like tails (H & E, ×80)

Physical examination revealed multiple, skin to tan-colored, flat-topped papules, 1 to 3 mm in diameter, on the forearm, thigh, neck, anterior chest, upper abdomen [Figures 1–4]. The lesions were bilateral, symmetrical, and had both follicular and nonfollicular distribution. No puncta or significant surface changes were noted and Darier's sign was negative. The remainder of the physical examination was unremarkable. A skin biopsy was obtained from a lesion in the forearm. Punch biopsy specimen revealed a normal epidermis overlying a dermis that was filled with multiple ducts embedded in a fibrous stroma. The ducts were lined by an inner layer of flattened epithelial cells. Some had a tadpole-like appearance due to the presence of a comma-like tail, which was formed by the cells projecting from one side of the duct into the stroma. Ductal lumina were filled with an amorphous material [Figure 5, Figure 6].

## Discussion

Syringoma is a benign adnexal tumor that derives from the intraepidermal portion of the eccrine sweat ducts. Syringomas first appear during puberty or the third or fourth decade.[1,4,5] Although the variety localized on the eyelid in middle-aged women is the most frequent, many other clinical variants are reported. Friedman and Butler proposed a classification, based on the clinical features and associations. This consisted of four principal clinical variants of syringoma: a localized form, a familial form, a form associated with Down's syndrome, and a generalized form that encompasses multiple and eruptive syringoma.[1] The eruptions are generally asymptomatic, although pruritus has been reported in some cases. The lesions are benign and may spontaneously resolve, or, more commonly, remain stable.[3]

Our case was a 28-year old woman who had lesions of generalized eruptive form. The patient presented with an eight-year history of a dermatosis. The lesions appeared on the forearm first, which was followed by successive eruptions on the face, chest, upper abdomen, thigh and neck respectively. The patient had mild pruritus during perspiration. There was no spontaneous resolution in the period of observation.

Treatment of syringoma is cosmetic. Therefore, it should not be confused with the above referred differential diagnosis. Options are abundant and generally unsatisfactory, as they are located in the dermis and often numerous. Physical techniques such as excision, electrocoagulation and liquid nitrogen cryotherapy and dermabrasion yield poor cosmetic results.[3,7,8] Oral isotretinoin and topical tretinoin and adapalene have been used, as well as ablative techniques such as the CO_2_ laser, with variable success; however, none eliminates the risk of recurrence and, therefore, treatment of syringoma is often frustrating.[9] Most of the literature suggests using carbon dioxide laser.[10–12] One study demonstrates good results with temporary tattooing, following Q-switched alexandrite laser.[13] Unfortunately, all surgical interventions result in scarring.[12]

Treatment of syringoma is cosmetic. They are abundant and generally unsatisfactory. Treatment modalities have included dermabrasion, various methods of excision, cryosurgery, electrodesiccation, chemical peeling, and oral and topical retinoids.[8–10] Successful treatment of facial syringomas with carbon dioxide laser also has been reported.[11] A recent report suggests the use of topical atropine to alleviate the pruritus in symptomatic eruptive syringoma.[12]
